# Insight into the Role of Nanoparticles Shape Factors and Diameter on the Dynamics of Rotating Water-Based Fluid

**DOI:** 10.3390/nano12162801

**Published:** 2022-08-15

**Authors:** Asia Ali Akbar, N. Ameer Ahammad, Aziz Ullah Awan, Ahmed Kadhim Hussein, Fehmi Gamaoun, ElSayed M. Tag-ElDin, Bagh Ali

**Affiliations:** 1 Department of Mathematics, University of the Punjab, Lahore 54590, Pakistan; 2 Department of Mathematics, Faculty of Science, University of Tabuk, P.O. Box 741, Tabuk 71491, Saudi Arabia; 3 Mechanical Engineering Department, College of Engineering, University of Babylon, Hilla 00964, Iraq; 4 College of Engineering, University of Warith Al-Anbiyaa, Karbala 56001, Iraq; 5 Department of Mechanical Engineering, College of Engineering, King Khalid University, Abha 61421, Saudi Arabia; 6 Faculty of Engineering and Technology, Future University in Egypt, New Cairo 11835, Egypt; 7 School of Mathematics and Statistics, Northwestern Polytechnical University, Xian 710072, China; 8 Faculty of Computer Science and Information Technology, Superior University, Lahore 54000, Pakistan

**Keywords:** stretching surface, magnetohydrodynamics, rotating Maxwell fluid, nanofluid, nanoparticles diameter

## Abstract

This article addresses the dynamic of three-dimensional rotating flow of Maxwell nanofluid across a linearly stretched sheet subject to a water-based fluid containing copper nanoparticles. Nanoparticles are used due to their fascinating features, such as exceptional thermal conductivity, which is crucial in modern nanotechnology and electronics. The primary goal of this comprehensive study is to examine the nanoparticles size and shape factors effect on the base fluid temperature. The mathematical model contains the governing equations in three dimensional partial differential equations form, and these equations transformed into dimensionless ordinary dimensional equations via suitable similarity transformation. The bvp4c technique is harnessed and coded in Matlab script to obtain a numerical solution of the coupled non-linear ordinary differential problem. It is observed that the greater input of rotating, Deborah number, and magnetic parameters caused a decline in the fluid primary and secondary velocities, but the nanoparticles concentration enhanced the fluid temperature. Further, a substantial increment in the nanofluid temperature is achieved for the higher nanoparticle’s diameter and shape factors.

## 1. Introduction

The flow over a stretching surface is a significant challenge in many engineering and technological processes [1,2,3], including the cooling of metallic plates, copper spiraling, aerodynamic extrusion of rubber and plastic covers, strengthening and tinning of copper wires, glass fiber and paper production, etc. Crane [4] was the first to observe the basic phenomenon of flowing on a linear expanding surface. Wang [5] investigated the viscid stream generated by a horizontally stretched surface, including suction and slipping phenomena. The magnetohydrodynamic (MHD) boundary layer stream of a Powell–Eyring nanofluid on a nonlinear expanding surface of varying width was scrutinized by Hayat et al. [6]. Majeed et al. [7] probed the heat transmission attributes of ferromagnetic viscous liquid flow over a stretching sheet with a linear speed, considering the influence of sucking and magnetic polarization. Awan et al., investigated various flows over the stretched surfaces and porous medium [8,9,10].

Magnetohydrodynamics (MHD) is a subfield of fluid dynamics that explores how an electrically conductive liquid moves in the presence of a magnetic field. Alfvén [11] was the first to identify that the passage of conducting liquid between magnetic field lines creates potential differences, allowing electric currents to pass. Hayat et al. [12] exposed the heat transport properties of a boundary layer stream across a permeable stretched surface under the influence of MHD and slipped constraints. Mabood et al. [13] developed the exact solutions for the MHD boundary layer stream of a thermally radiating viscous liquid over an exponentially stretched surface. The numerical findings for the laminar stream over a vertically stretched sheet involving magnetism and viscosity dissipation were probed by Alarifi et al. [14]. Ali et al. [15,16] have studied the effects of MHD on different types of flows. The Lorentz force is very helpful in the enhancement of the fluid temperature [17,18], it is due to the Lorentz force which caused the fluid velocity to recede [19,20].

The applications of non-Newtonian fluids cover a wide range of fundamental challenges in pharmaceutical, crude oil, polymer, and food production industries [21,22]. Viscoelastic fluids are non-Newtonian fluids in which the imposed tension distribution is non-linearly related to velocity gradient. The strain rate steadily decreases when shear tension is removed from these fluids. This is referred to as stress relaxation. Furthermore, relaxation time is the time it takes the fluid to recover somewhat elastically when stress is removed. The upper convected Maxwell (UCM) liquid model is the most basic model of viscoelasticity. Exact solutions for the rotational stream of a modified Burgers’ fluid over cylindrical regions were proposed by Jamil and Fetecau [23]. Kamran et al. [24] investigated the unstable rotating flow of fractional Oldroyd-B liquid between two infinite concentric cylinders. Mustafa [25] utilized the Cattaneo–Christov thermal transport model to scrutinize Maxwell liquid’s rotational stream confined by a sheet. The three-dimensional (3D) nanofluid stream of a rotational Maxwell fluid over an exponentially enlarged surface was probed by Hayat et al. [26]. Ahmed et al. [27] scrutinized the MHD swirled stream and thermal transport in Maxwell liquid propelled by two concentrically spinning discs with varying heat conductivity. Waqas et al. [28] revealed the effect of mixed convection over a Maxwell nanofluid stream caused by motile organisms swimming across a vertically revolving cylinder.

Based on recent achievements in nanotechnology, nanomaterial enrichment is thought to be very efficient in boosting the thermal conductivity of base solvents. Nanofluids are designed colloids comprised of the base fluid and nanoparticles. Choi and Eastman [29] are the pioneers who realized that base fluids having less thermal conductivity are not efficient for better heat transfer. Das [30] examined the numerical solutions for the boundary layer flow of nanoliquid across a nonlinear porous extending surface with partial slip effects. After that, much research has been conducted on different nanofluid streams [31,32,33,34,35]. Thermal conductance and specific heat capacity at various degrees of nanoparticle concentration, nanoparticle size, diameter, and inter-particle spacing are some of the thermal attributes. Namburu et al. [36] discovered that raising the radius of $SiO_{2}$ nanoparticles in ethylene glycol and water reduces the viscosity of the nanoliquid. Adopting a two-phase technique, Akbarinia and Laur [37] exposed the impact of nanoparticle diameter on a laminar nanoliquid stream in a curvy tube. It was discovered that when the shape of the nanoparticles deviates significantly from the spherical, a significant increase in effective thermal conductance can be obtained. The thermal conductance and mechanical impact of uniformly scattered silicon carbide nanoparticles of a mean size of 170nm in water were inspected by Singh et al. [38]. Ashraf et al. [39] probed the role of nanoparticle size and interface depth. Sowmya et al. [40] examined the thermal efficiency of the nanofluid stream through a permeable fin containing nanoparticles of molybdenum disulfide that had different shapes.

Motivated by the above-cited literature, we concluded that the present work on the rotational 3-D Maxwell nanofluid flow caused by a linearly stretching surface subject to MHD and partial slip has not been studied yet. The purposes of this investigation are: (i) to observe the steady 3-D flow of Maxwell fluid in a rotational frame with partial slip and MHD; and (ii) to enhance the heat transfer rate of nanofluid using nanoparticles of different shapes and diameters. The mathematical model is formed under certain assumptions, and the governed PDEs are transformed into dimensionless ODEs via similarity analysis. The resulting ODEs are tackled in a MATLAB script with the bvp4c technique. The significant findings for the primary and secondary velocities, temperature, skin frictions, and wall heat transfer coefficient are portrayed via graphs and analyzed through tables. The validity of the numerical technique is validated by comparative tables with the existing research work. This numerical study is relevant to electrochemical procedures, microelectronics, and heat exchange, etc.

## 2. Mathematical Analysis and Flow Geometry

**Figure 1:**
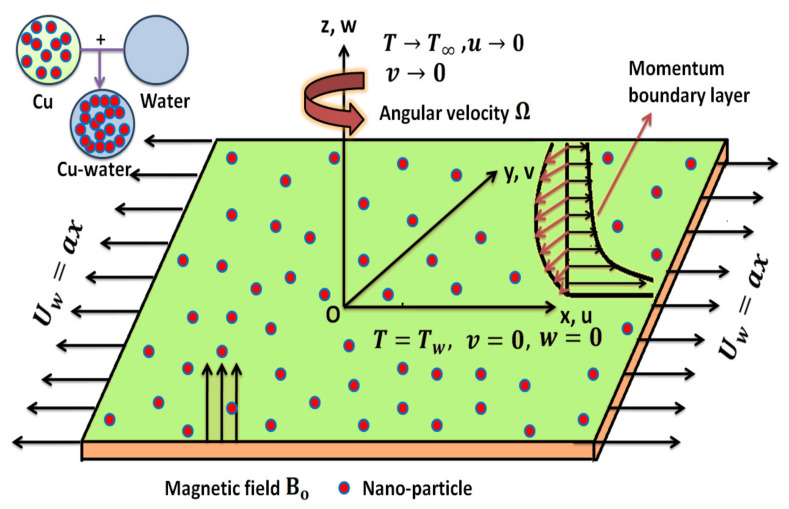
Geometric configuration and coordinate scheme.

The steady, incompressible, rotating, laminar, and 3−D Maxwell nanoliquid stream across a linearly stretched surface is analyzed. The nanofluid contains solid nanoparticles of Cu and water as the base fluid. At z=0, the sheet touches the plane and the motion of fluid is maintained in the region z>0 (see Figure 1). The sheet is stretched along the *x*-axis with a stretching velocity $U_{w}\left(x\right)$ that is proportional to the stretching distance from the origin. Furthermore, the fluid spins constantly around the *z*-axis with a fixed angular velocity $\tilde{\Omega }$ and the angular velocity is $\Omega =[0,0,\tilde{\Omega }]$. The whole system is rotating at velocity angular $\tilde{\Omega }$ along the *z*-direction which is orientated transverse to the plane of the sheet (xy-plane). An MHD effect is considered by applying a uniform magnetic field $\left(B_{o}\right)$ normally to the sheet. $T_{w}$ represents the temperature of the stretched sheet which remains constant, and $\left(T_{w}>T_{\infty }\right)$ where $T_{\infty }$ is the temperature far away from the sheet. The velocity vector for the current flow situation is supposed as $\tilde{V}=\left[u\left(x,y,z\right),v\left(x,y,z\right),w\left(x,y,z\right)\right]$. The induced magnetic field is neglected because its strength is small in magnitude.

The conservation of fluid’s mass, momentum, and energy equations for the considered flow phenomena are stated as [41,42,43]:

(1) $$\begin{array}{cc} \partial_{x}u+\partial_{y}v+\partial_{z}w & =0, \end{array}$$

(2) $$\begin{array}{cc} u\partial_{x}u+v\partial_{y}u+w\partial_{z}u & =2\tilde{\Omega }v+\frac{\mu_{nf}}{\rho_{nf}}(\partial_{xx}\;u+\partial_{yy}\;u+\partial_{zz}\;u)-\frac{\sigma_{nf}}{\rho_{nf}}B_{o}^{2}u\\ & -\lambda_{1}\left\{\begin{array}{c} u^{2}\partial_{xx}u+v^{2}\partial_{yy}u+w^{2}\partial_{zz}u\\ +2vw\partial_{y}\partial_{z}u+2uv\partial_{x}\partial_{y}u+2uw\partial_{x}\partial_{z}u\\ -2\tilde{\Omega }\left(u\partial_{x}v+v\partial_{y}v+w\partial_{z}v\right)+2\tilde{\Omega }\left(v\partial_{x}u-u\partial_{y}u\right) \end{array}\right\}, \end{array}$$

(3) $$\begin{array}{cc} u\partial_{x}v+v\partial_{y}v+w\partial_{z}v & =-2\tilde{\Omega }u+\frac{\mu_{nf}}{\rho_{nf}}(\partial_{xx}\;v+\partial_{yy}\;v+\partial_{zz}\;v)-\frac{\sigma_{nf}}{\rho_{nf}}B_{o}^{2}v\\ & -\lambda_{1}\left\{\begin{array}{c} u^{2}\partial_{xx}v+v^{2}\partial_{yy}v+w^{2}\partial_{zz}v\\ +2vw\partial_{y}\partial_{z}v+2uv\partial_{x}\partial_{y}v+2uw\partial_{x}\partial_{z}v\\ +2\tilde{\Omega }\left(u\partial_{x}u+v\partial_{y}u+w\partial_{z}u\right)+2\tilde{\Omega }\left(v\partial_{x}v-u\partial_{y}v\right) \end{array}\right\}, \end{array}$$

(4) $$\begin{array}{cc} u\partial_{x}w+v\partial_{y}w+w\partial_{z}w & =\frac{\mu_{nf}}{\rho_{nf}}(\partial_{xx}\;w+\partial_{yy}\;w+\partial_{zz}\;w), \end{array}$$

(5) $$\begin{array}{cc} u\partial_{x}T+v\partial_{y}T+w\partial_{z}T & =\frac{k_{nf}}{{\left(\rho C_{p}\right)}_{nf}}\;(\partial_{xx}\;T+\partial_{yy}\;T+\partial_{zz}\;T). \end{array}$$

Here, (u,v,w) are the (x,y,z) constituents of velocity, $B_{o}$ is the applied magnetism, $\rho_{nf},\mu_{nf},\sigma_{nf}$ are the density, dynamic viscosity, and electrical conductivity of nanofluid, respectively. Furthermore, $\lambda_{1}$ is the relaxation time of the Maxwell fluid, ${\left(\rho C_{p}\right)}_{nf}$ is thermal conductivity of nanofluid, *T* denotes the fluid’s temperature, and $k_{nf}$ is the thermal conductivity of nanofluid. The no-slip boundary condition was only appropriate to macro-scale structures and was not suitable to micro and nano-scale structures [44]. Beavers and Joseph [45] pioneered the study of slip boundary condition. The temperature of $\left(Cu-H_{2}O\right)$ and $\left(TiO_{2}-H_{2}O\right)$ nanofluids was raised by the slip parameter [46]. The above elaborated fluid mathematical model boundary conditions are as follows [47]:

(6) $$\begin{array}{c} \left.\begin{array}{cc} u & =U_{w}+L\frac{\partial u}{\partial z},\;\;\;\;\;T_{w}=T,\;\;\;\;w=0,\;\;\;\;0=v,\;\;\;\;\mathrm{for}\;\;z\to 0\\ T & \to T_{\infty },\;\;\;\;v\to 0,\;\;\;\;u\to 0,\;\;\;\;\mathrm{for}\;\;z\to \infty . \end{array}\right\} \end{array}$$

Here, $U_{w}=ax$ is the surface stretching velocity along x–axis, *L* denotes the slip distance, and *a* is the rate of surface extension.

The relation between nanofluid and base fluid viscosity is given by following Gosukonda et al. [48]:

(7) $$\begin{array}{c} \frac{\mu_{nf}}{\mu_{f}}=1+\frac{5}{2}\phi_{s}+\frac{9}{2}\left[\frac{1}{\frac{h}{d_{n}}\left(2+\frac{h}{d_{n}}\right)\left(1+\frac{h}{d_{n}}\right)}\right], \end{array}$$

where $\phi_{s}$ is the solid particle concentration, $d_{n}$ depicts the nanoparticle’s radius, and *h* is the inter-particle distance. The shape factor $\left(s_{p}\right)$ is expressed as the ratio of a non-spherical nanoparticle’s surface area $\left(A^{\prime }\right)$ to that of a spherical nanoparticle’s (A), when both nanoparticles have the same volume [49], i.e., $s_{p}=\frac{A^{\prime }}{A}$.

The other properties of nanofluid are stated as follows [50,51]:

(8) $$\begin{array}{c} \rho_{nf}=\rho_{f}\left[\left(1-\phi_{s}\right)+\phi_{s}\frac{\rho_{s}}{\rho_{f}}\right],\;\;\;\;\;{\left(\rho C_{p}\right)}_{nf}={\left(\rho C_{p}\right)}_{f}\left[\left(1-\phi_{s}\right)+\phi_{s}\frac{{\left(\rho C_{p}\right)}_{s}}{{\left(\rho C_{p}\right)}_{f}}\right], \end{array}$$

(9) $$\begin{array}{c} \frac{k_{nf}}{k_{f}}=\left[\frac{k_{s}+k_{f}\left(s_{p}-1\right)-\phi_{s}\left(s_{p}-1\right)\left(k_{f}-k_{s}\right)}{k_{s}+k_{f}\left(s_{p}-1\right)+\phi_{s}\left(k_{f}-k_{s}\right)}\right],\;\;\;\;\frac{\sigma_{nf}}{\sigma_{f}}=\left[1+\frac{3\phi_{s}\left(\frac{\sigma_{s}}{\sigma_{f}}-1\right)}{\left(\frac{\sigma_{s}}{\sigma_{f}}+2\right)-\phi_{s}\left(\frac{\sigma_{s}}{\sigma_{f}}-1\right)}\right]. \end{array}$$

**Figure 2:**
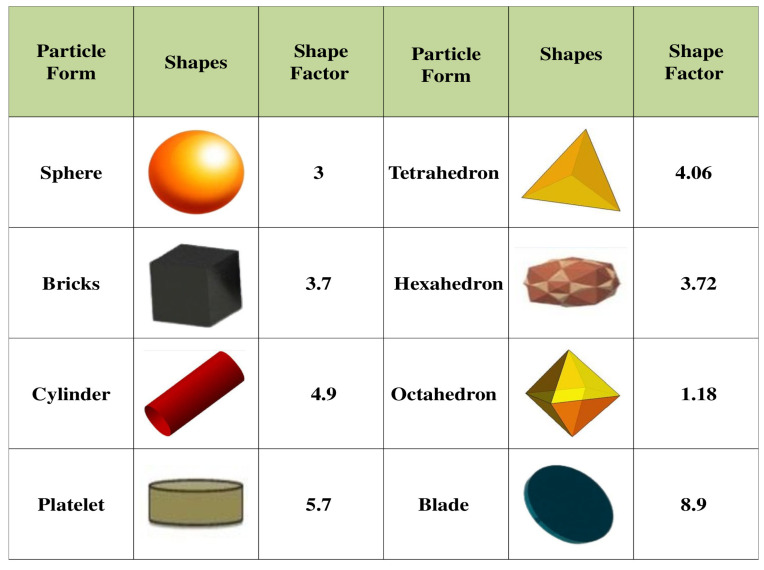
Shape of nanoparticles along with shape factor [53,54,55].

Here, $\rho_{f}$ is the base liquid density, $\rho_{s}$ is density of solid nanoparticle, ${\left(\rho C_{p}\right)}_{f}$ is the volumetric thermal capability of base fluid, ${\left(\rho C_{p}\right)}_{s}$ denotes the volumetric temperature ability of nanoparticle, $k_{f}$ denotes the heat conductivity of base fluid, $s_{p}$ is the shape factor for the nanoparticle, $k_{s}$ is the heat conductivity of the nanoparticle, $\sigma_{f}$ depicts the electric conductance of the base liquid, and $\sigma_{s}$ is the electric conductance of the solid nanoparticle. The thermo-physical physical properties of nanoparticles are described in Table 1. Figure 2 represents different shapes of nanoparticles along with shape factors. The following similarity transformation is applied to simplify the analysis [52]:

(10) $$\begin{array}{c} u=xaf^{\prime }\left(\xi \right),\;\;\;\;v=h\left(\xi \right)xa,\;\;\;\;w=-\sqrt{a\nu_{f}}f\left(\xi \right),\;\;\;\;\theta \left(\xi \right)=\frac{T-T_{\infty }}{T_{w}-T_{\infty }},\;\;\;\;\xi =z\sqrt{\frac{a}{\nu_{f}}}. \end{array}$$

Here ξ is the dimensionless constraint, $f^{\prime }\left(\xi \right)$ denotes the dimensionless velocity profile along x–axis, h(ξ) is velocity profile along y–axis, and θ(ξ) is dimensionless temperature profile. Employing the Equation (10), continuity equation is satisfied, and Equations (2)–(5) are transformed into the dimensionless ODEs utilizing Equations (7)–(10) as follows:

(11) $$\begin{array}{c} \frac{E_{1}}{E_{2}}f^{\prime \prime \prime }+2\lambda \left(h-\beta fh^{\prime }\right)+\beta \left(-f^{\prime \prime \prime }f^{2}+2f^{\prime }ff^{\prime \prime }\right)+ff^{\prime \prime }-{\left(f^{\prime }\right)}^{2}-\frac{E_{3}}{E_{2}}Mf^{\prime }=0, \end{array}$$

(12) $$\begin{array}{c} \frac{E_{1}}{E_{2}}h^{\prime \prime }-f^{\prime }h+fh^{\prime }-2\lambda \left[\beta \left({f\prime }^{2}-f^{\prime \prime }f+h^{2}\right)+f^{\prime }\right]+\beta \left(2h^{\prime }ff^{\prime }-h^{\prime \prime }f^{2}\right)-\frac{E_{3}}{E_{2}}Mh=0, \end{array}$$

(13) $$\begin{array}{c} \frac{E_{4}}{E_{5}}\;\theta^{\prime \prime }+Prf\theta^{\prime }=0, \end{array}$$

where

$$\begin{array}{c} E_{1}=1+\frac{5}{2}\phi_{s}+\frac{9}{2}\left[\frac{1}{\frac{h}{d_{n}}\left(2+\frac{h}{d_{n}}\right)\left(1+\frac{h}{d_{n}}\right)}\right],\;\;\;E_{2}=1-\phi_{s}+\phi_{s}\frac{\rho_{s}}{\rho_{f}},\;\;\;\lambda =\frac{\tilde{\Omega }}{a},\\ E_{3}=\left[1+\frac{3\phi_{s}\left(\frac{\sigma_{s}}{\sigma_{f}}-1\right)}{\left(\frac{\sigma_{s}}{\sigma_{f}}+2\right)-\phi_{s}\left(\frac{\sigma_{s}}{\sigma_{f}}-1\right)}\right],\;\;\;E_{4}=\left[\frac{k_{s}+k_{f}\left(s_{p}-1\right)-\phi_{s}\left(s_{p}-1\right)\left(k_{f}-k_{s}\right)}{k_{s}+k_{f}\left(s_{p}-1\right)+\phi_{s}\left(k_{f}-k_{s}\right)}\right],\\ E_{5}=1-\phi_{s}+\phi_{s}\frac{{\left(\rho C_{p}\right)}_{s}}{{\left(\rho C_{p}\right)}_{f}},\;\;\;\beta =\lambda_{1}a,\;\;\;M=\frac{\sigma_{f}B_{o}^{2}}{\rho_{f}a},\;\;\;Pr=\frac{\nu_{f}{\left(\rho C_{p}\right)}_{f}}{k_{f}}. \end{array}$$

The dimensionless parameters are λ,β,M, and Pr which respectively denote the rotational parameter, Deborah number, magnetic parameter, and Prandtl number. The dimensionless boundary constraints are obtained by employing Equation (10) in Equation (6).

(14) $$\begin{array}{c} \left.\begin{array}{c} \mathrm{At}\;\;\;\xi \to 0;\;\;\;\;K_{1}f^{\prime \prime }\left(\xi \right)+1=f^{\prime }\left(\xi \right),\;\;\;0=h\left(\xi \right),\;\;\;0=f\left(\xi \right),\;\;\;\theta \left(\xi \right)=1,\\ \mathrm{At}\;\;\;\xi \to \infty ;\;\;\;0=f^{\prime }\left(\xi \right),\;\;\;0=h\left(\xi \right),\;\;\;0=\theta \left(\xi \right), \end{array}\right\} \end{array}$$

where $K_{1}=L{\left(\frac{a}{\nu_{f}}\right)}^{1/2}$ denotes the dimensionless slip parameter.

Physical variables of interest, such as skin frictions along x− and y− axes $\left(\tilde{C}f_{x},\tilde{C}f_{y}\right)$, and Nusselt number $\left(Nu_{x}\right)$ are given as:

(15) $$\begin{array}{c} \tilde{C}f_{x}=\frac{{\tilde{\tau }}_{xz}}{\rho_{nf}U_{w}^{2}},\;\;\;\;\;\;\tilde{C}f_{y}=\frac{{\tilde{\tau }}_{yz}}{\rho_{nf}U_{w}^{2}},\;\;\;\;\;\;Nu_{x}=\frac{x\tilde{q_{w}}}{k_{f}\left(T_{w}-T_{\infty }\right)}, \end{array}$$

where $\tilde{q_{w}}$ denotes heat influx and $\left({\tilde{\tau }}_{xz},{\tilde{\tau }}_{yz}\right)$ signify the wall shear stresses.

(16) $$\begin{array}{c} {\tilde{\tau }}_{xz}=\mu_{nf}\left(\frac{\partial w}{\partial x}+\frac{\partial u}{\partial z}\right){|}_{z=0},\;\;\;\;{\tilde{\tau }}_{yz}=\mu_{nf}\left(\frac{\partial w}{\partial y}+\frac{\partial v}{\partial z}\right){|}_{z=0},\;\;\;\;\tilde{q_{w}}=-k_{nf}\left(\frac{\partial T}{\partial z}\right){|}_{z=0}. \end{array}$$

By using Equations (7)–(10) in Equation (15), the dimensionless forms of physical quantities are stated as:

(17) $$\begin{array}{c} \sqrt{Re_{x}}\tilde{C}f_{x}=\frac{E_{1}}{E_{2}}f^{\prime \prime }\left(0\right),\;\;\;\;\;\sqrt{Re_{x}}\tilde{C}f_{y}=\frac{E_{1}}{E_{2}}h^{\prime }\left(0\right),\;\;\;\;\;\frac{Nu_{x}}{\sqrt{Re_{x}}}=-\frac{k_{nf}}{k_{f}}\theta^{\prime }\left(0\right), \end{array}$$

where $\sqrt{Re_{x}}=\sqrt{\frac{ax^{2}}{\nu_{f}}}$ is the local Reynolds number.

## 3. Numerical Procedure

The system of coupled ODEs (11–13), as well as the relevant boundary constraints (14), are difficult to address analytically because of the non-linearity of the equations. Several numerical approaches are utilized in MATLAB to solve such flow problems. The one of the most common and powerful method is MATLAB built-in technique bvp4c. The solution method is stated as:

$$\begin{array}{c} y\left(1\right)=f;\;\;f^{\prime }=y\left(2\right);\;\;y\left(3\right)=f^{\prime \prime };\;\;f^{\prime \prime \prime }=yy1;\;\;h=y\left(4\right);\\ h^{\prime }=y\left(5\right);\;\;h^{\prime \prime }=yy2;\;\;\theta =y\left(6\right);\;\;\theta^{\prime }=y\left(7\right);\;\;\theta^{\prime \prime }=yy3. \end{array}$$

The non-linear ODEs are converted into the following 1st order ODEs using the above substitutions.

(18) $$\begin{array}{cc} yy1 & ={\left(\frac{E_{1}}{E_{2}}-y\left(1\right)*\beta *y\left(1\right)\right)}^{-1}*(-y\left(1\right)*y\left(3\right)+y\left(2\right)*y\left(2\right)-\beta *2*y\left(1\right)*y\left(2\right)*y\left(3\right)\\ \; & +\frac{E_{3}}{E_{2}}*M*y\left(2\right)-2*\lambda *\left(y(4)-2*y(1)*\beta *y(5)\right)); \end{array}$$

(19) $$\begin{array}{cc} yy2 & ={\left(\frac{E_{1}}{E_{2}}-y\left(1\right)*\beta *y\left(1\right)\right)}^{-1}*(-y\left(1\right)*y\left(5\right)+\frac{E_{3}}{E_{2}}*M*y\left(4\right)+y\left(4\right)*y\left(2\right)+2*\lambda *\\ & (y\left(2\right)+(-y(1)*y(3)+y(2)*y(2)+y(4)*y(4))*\beta )-2*y\left(1\right)*\beta *y\left(5\right)*y\left(2\right)); \end{array}$$

(20) $$\begin{array}{cc} yy3 & =-{\left(\frac{E_{4}}{E_{5}}\right)}^{-1}*y\left(1\right)*Pr*y\left(7\right). \end{array}$$

The boundary constraints are given as:

(21) $$\begin{array}{c} y0\left(2\right)-(1+K_{1}*y0\left(3\right));\;\;\;y0\left(1\right);\;\;\;y0\left(4\right);\;\;\;y0\left(6\right)-1;\;\;\;yinf\left(2\right);\;\;\;yinf\left(4\right);\;\;\;yinf\left(6\right). \end{array}$$

## 4. Results and Discussion

**Figure 3:**
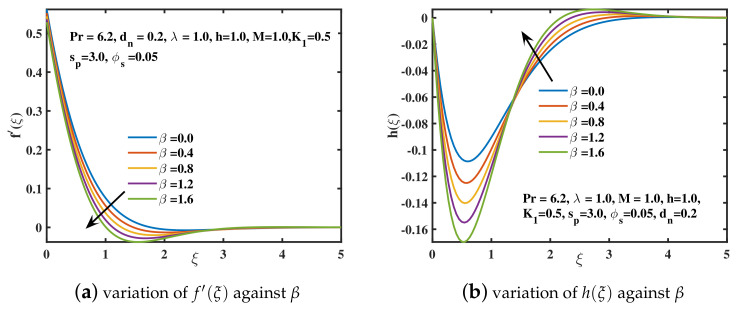
The $f^{\prime }\left(\xi \right)$ and h(ξ) curves for distinct Deborah number (β) values.

**Figure 4:**
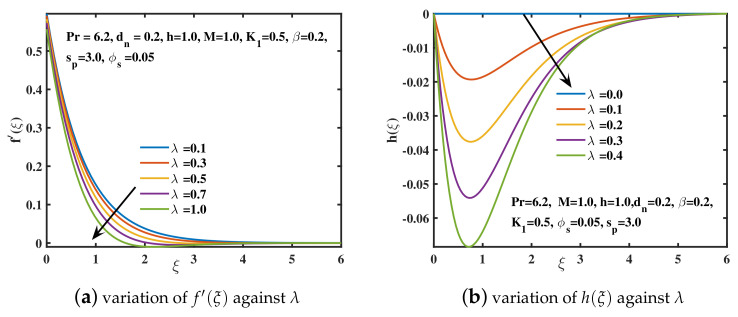
The $f^{\prime }\left(\xi \right)$ and h(ξ) curves for distinct rotating parameter (λ) values.

**Figure 5:**
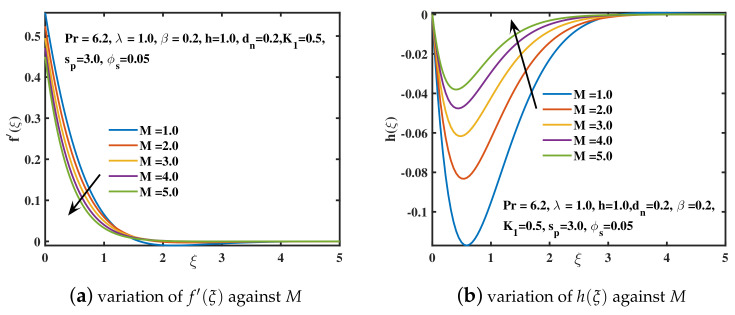
The $f^{\prime }\left(\xi \right)$ and h(ξ) curves for distinct magnetic parameter *M* values.

**Figure 6:**
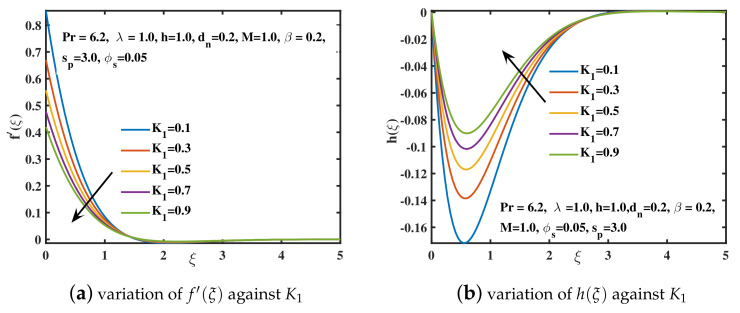
The $f^{\prime }\left(\xi \right)$ and h(ξ) curves for distinct slip parameter $K_{1}$ values.

**Figure 7:**
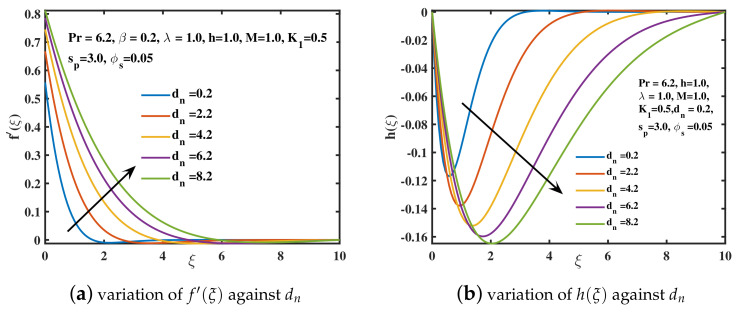
The $f^{\prime }\left(\xi \right)$ and h(ξ) curves for distinct diameter $d_{n}$ values.

**Figure 8:**
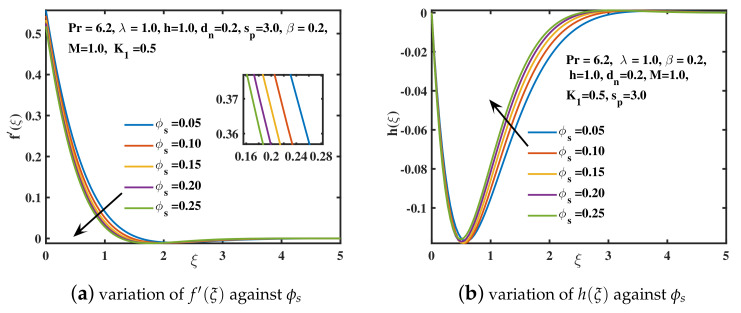
The $f^{\prime }\left(\xi \right)$ and h(ξ) curves for distinct $\phi_{s}$ values.

**Figure 9:**
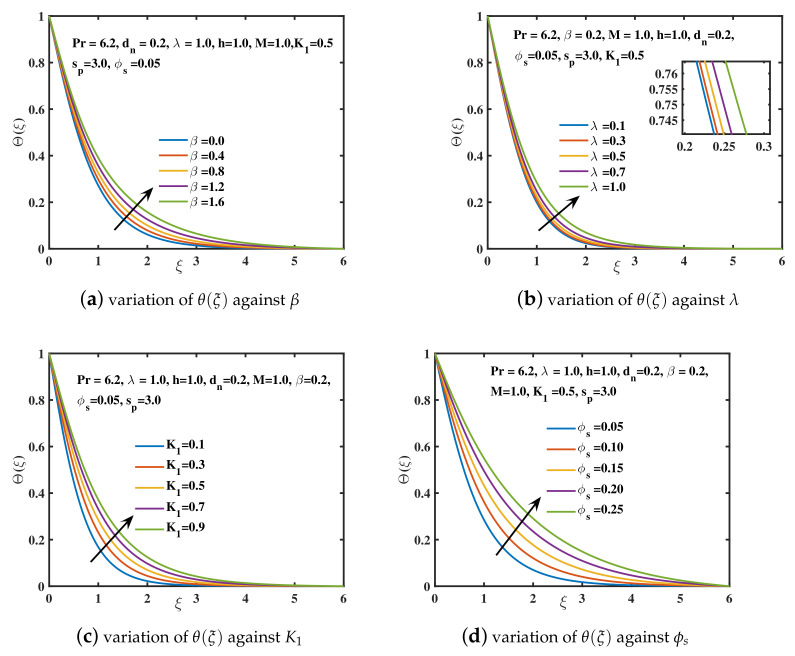
The effect of $\beta ,\lambda ,K_{1},\mathrm{and}\;\phi_{s}$ on temperature curve.

**Figure 10:**
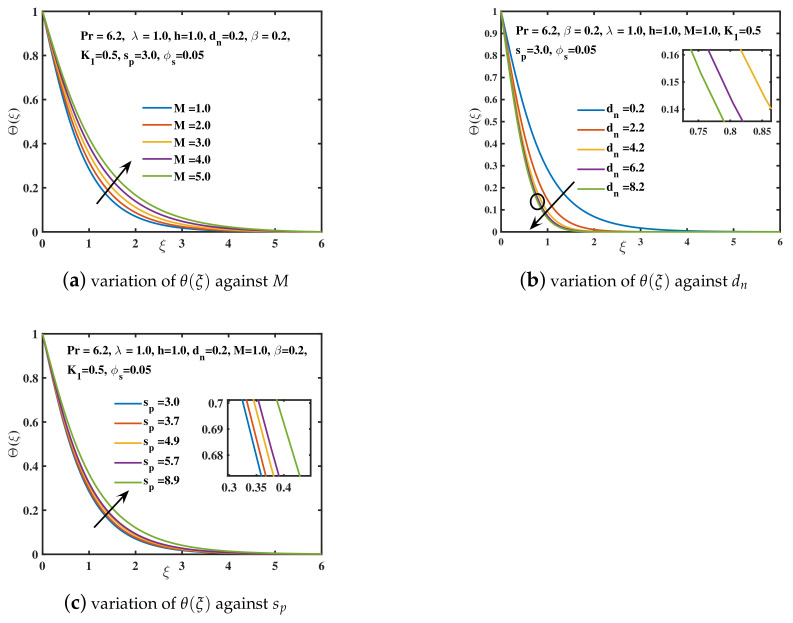
The effect of Magnetic parameter *M*, nanoparticle diameter $d_{n}$, and shape factor $s_{p}$ on temperature profile.

In the current study, the flow phenomena arising due to the linear stretching of an expanding sheet in a rotational upper-convected Maxwell nanofluid is studied. The computational outcomes for the current work are evaluated by solving the dimensionless ODEs (11–13) along with the boundary and initial constraints (14). The estimation for the current analysis is settled by using the specified values for parameters: λ=1.0,β=0.2,M=1.0,$K_{1}=0.5,\phi_{s}=0.05,d_{n}=0.2,s_{p}=3.0,h=1.0$. The bvp4c technique in MATLAB software is used to get the numeric results. We used Table 2 and Table 3 to compare our findings to previously published studies. A significant coincidence has been achieved that proves the validity of the bvp4c method. The impacts of various parameters on velocity curves $f^{\prime }\left(\xi \right)$, h(ξ) and temperature curve θ(ξ) are elaborated through Figure 3, Figure 4, Figure 5, Figure 6, Figure 7, Figure 8, Figure 9 and Figure 10.

Figure 3a,b involves the dimensionless velocity profiles along *x* and *y*-axis for the various values of β (Deborah number). Deborah number is the quotient of fluid’s relaxation time and the time of observation. It is used to assess the fluidity of the substances under specified flow regimes. For the smaller values of Deborah number, the fluid flow becomes purely viscous. Nevertheless, for the higher values of β, the fluid acts like solid material. So, as β boosts up, the velocity curve $f^{\prime }\left(\xi \right)$ declines, and far outside of the boundary layer, the motion of fluid diminishes. This is because of the reason that the viscoelastic impacts retard the flow along *x*-axis, and ultimately give shorter boundary layer. The secondary velocity profile h(ξ), or velocity along *y*-direction, is basically caused by the rotational frame, because the sheet is extended only along the *x*-axis. However, the velocity h(ξ) falls in magnitude away from the stretched sheet, while the opposing behavior is examined in the vicinity of the sheet. Figure 4a,b depicts the significance of the rotational parameter λ on the primary and secondary velocities. The case λ=0.0 represents the motion in a non-rotational framework. As $\lambda =\frac{\tilde{\Omega }}{a}$ enhances, which is the quotient of rotation to stretching rate, $f^{\prime }\left(\xi \right)$ declines. Physically, the stretching rate dominates the rotation rate for higher values of λ. As a result, the stronger rotational impacts offer resistance to fluid flow in the *x*-direction, causing the boundary layer thickness to decrease. The magnitude of velocity h(ξ) accelerates for the higher values of λ along the negative *y*-axis. An oscillatory pattern is observed for the velocity curve h(ξ) as reported by [41].

Figure 5a,b reflects the behavior of $f^{\prime }\left(\xi \right)$ and h(ξ) for the progressive values of magnetic parameter *M*. It is analyzed that the velocity curve $f^{\prime }\left(\xi \right)$ decreases for increased *M* values. The reduction in velocity is induced by an elevation in resisting force called the Lorentz force, which is caused by the interplay of magnetic and electrical fields. Back flow for $f^{\prime }\left(\xi \right)$ is prevented by stretching the sheet along the *x*-axis. The velocity $f^{\prime }\left(\xi \right)$ is maximal at ξ=0 and it diminishes far away from the surface of sheet. Figure 5b depicts that the backflow dominates the velocity along the *y*-axis and h(ξ) acquires negative values. The secondary velocity enhances near the sheet and then tends to diminish as shown by [43]. The influence of the slip factor $K_{1}$ on velocities $f^{\prime }\left(\xi \right)$ and h(ξ) is examined in Figure 6a,b. The velocity curve $f^{\prime }\left(\xi \right)$ and the amplitude of the secondary velocity curve h(ξ) show a declining trend. Physically, as the slip parameter $K_{1}$ enhances, the stretching speed and the speed of liquid near the sheet doesn’t meet. Hence, the velocity $f^{\prime }\left(\xi \right)$ declines and tends to become zero far off the sheet. Similar behavior is observed for the amplitude of velocity h(ξ) as explored by [42].

The significance of rising the nanoparticle diameter $d_{n}$ over the velocities $f^{\prime }\left(\xi \right)$ and h(ξ) is explored in Figure 7a,b. As the diameter $d_{n}$ (nanoparticle surface to volume ratio) boosts up, the flow is accelerated in the *x*-direction and boundary layer thickness is upgraded. It is because enhancing the diameter of nanoparticles in water causes a decrement in viscosity of nanofluid as explored by [51]. The magnitude of velocity in the y–direction is also enhanced by increasing the diameter of nanoparticles. The increasing values of nanoparticle concentration $\phi_{s}$ in the nanofluid depreciates the velocity $f^{\prime }\left(\xi \right)$ as depicted in Figure 8a. This is due to the collision of nanoparticles which offers retardation to fluid motion. As a result, boundary layer width diminishes for higher values of $\phi_{s}$. A similar fashion is portrayed in Figure 8b for the magnitude of *y*-direction velocity h(ξ). Figure 9a is portrayed for different values of Deborah number β against the temperature θ(ξ) of nanofluid. It has been visualized that the temperature of nanofluid is augmented for larger values of Deborah number. A thicker temperature boundary layer is observed in case of higher values of β. From Figure 9b it is visualized that the temperature of the nanofluid is augmented with the increment in rotational parameter λ. Technically, the higher the rotating parameter, the more kinetic energy the nanofluid has, which raises the temperature θ(ξ) as probed by [41]. In Figure 9c the temperature curve θ(ξ) is plotted against the slip parameter $K_{1}$. The nanofluid’s temperature θ(ξ) and thermal penetrating depth incremented for the higher values of slip coefficient. Figure 9d reflected that by augmenting the nanoparticle concentration $\phi_{s}$, the temperature of the nanoliquid rises. Physically, when the concentration $\phi_{s}$ of the nanoparticles is boosted, the collisions among nanoparticles are increased. Because of these collisions, the thermal conductance of the nanofluid improves. Thus, energy is released by nanoparticles in the form of heat causing the temperature to enhance [42].

The rising values of magnetic *M* tend to enhance the temperature θ(ξ) of the nanoliquid as observed in Figure 10a. The retardation of fluid caused by the Lorentz force in the boundary layer emanated in heat dissipation. As a result of this extra generated heat, the nanofluid’s temperature, as well as temperature boundary layer size expands with the rising values of *M*. Figure 10b reveals the consequence of $d_{n}$ on the θ(ξ) curve. With enhancing the diameter of nanoparticles, the temperature of the nanoliquid declines, and the thinner temperature boundary layer is examined. Further, it is probed that by using differently shaped nanoparticles, the thermal conductivity of the nanofluid is improved. It is observed in Figure 10c that the temperature is highest in the case of blade shape and a minimum in the case of spherical shape nanoparticles of copper.

The trend of skin friction coefficients $\left({\left(Re_{x}\right)}^{0.5}\tilde{C}f_{x},{\left(Re_{x}\right)}^{0.5}\tilde{C}f_{y}\right)$ and local Nusselt number $Nu_{x}{\left(Re_{x}\right)}^{-0.5}$ is analyzed in Table 4 for the different values of parameters. The increasing values of the rotating parameter λ reveals a progressive fashion for the magnitude of both the primary and secondary skin frictions. Furthermore, all negative $\tilde{C}f_{x}{\left(Re_{x}\right)}^{0.5}$ values suggest a reversing of the primary stream near the surface. However, the reduced fashion is observed for the Nusselt number $Nu_{x}{\left(Re_{x}\right)}^{-0.5}$. The augmented values of Deborah number β show that the magnitude of skin friction coefficients $\left({\left(Re_{x}\right)}^{0.5}\tilde{C}f_{x},{\left(Re_{x}\right)}^{0.5}\tilde{C}f_{y}\right)$ is elevated, but the Nusselt number, $Nu_{x}{\left(Re_{x}\right)}^{-0.5}$, is diminished.

**Figure 11:**
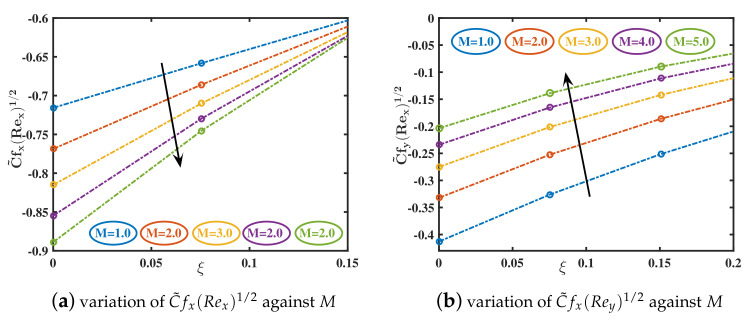

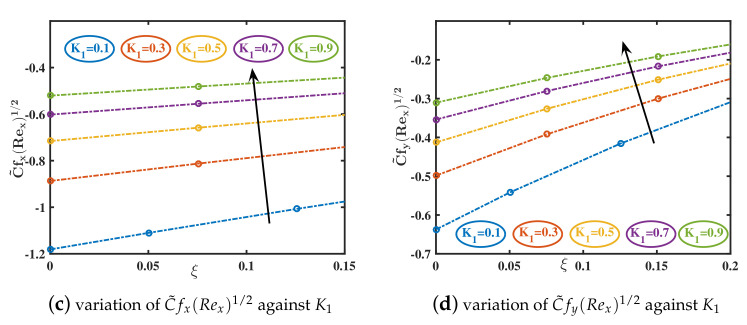
Variations in $\tilde{C}f_{x}{\left(Re_{x}\right)}^{1/2}$ and $Cf_{y}{\left(Re_{x}\right)}^{1/2}$ trend for different values of *M* and $K_{1}$.

**Figure 12:**
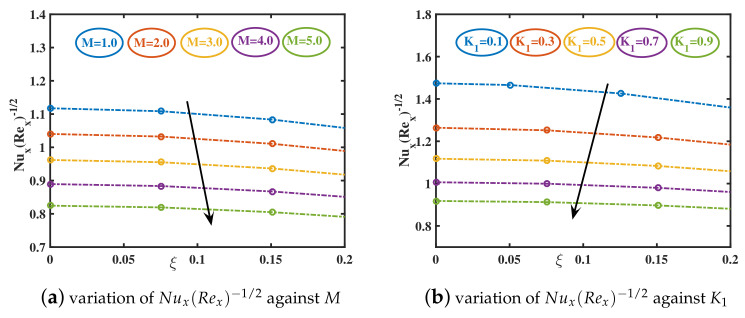
Variations in $Nu_{x}{\left(Re_{x}\right)}^{-1/2}$ trend for different amounts of *M* and $K_{1}$.

The primary skin friction, ${\left(Re_{x}\right)}^{0.5}\tilde{C}f_{x}$, is observed to grow negatively as the magnetic coefficient rises. This signifies that when a steadily rising magnetic field is applied, primary flow is intensified but reversed. Conversely, the secondary skin friction, ${\left(Re_{x}\right)}^{0.5}\tilde{C}f_{y}$, is observed to be damped in value as the magnetic field increases (see Figure 11a,b), Increasing the magnetic parameter, which refers to a significantly stronger axial magnetic field and an increment in magnetic Lorentz resisting forces in the *x* and *y*-directions has a significant impact on the magnitudes of both primary and secondary skin friction coefficients for all axial coordinate values. The local Nusselt number declines for the growing values of *M*. Increasing the slip parameter $K_{1}$ recedes the magnitude of $\left({\left(Re_{x}\right)}^{0.5}\tilde{C}f_{x},{\left(Re_{x}\right)}^{0.5}\tilde{C}f_{y}\right)$(see Figure 11c,d) and Nusselt number. This is because the slip coefficient enhances the surface friction causing the wall heat transmission rate to increase (see Figure 12a,b).

The rising values of the $\phi_{s}$ boost the primary and secondary skin frictions. The addition of nanoparticles in base liquid thickens the fluid and generate resistance. As a result, the Nusselt number $Nu_{x}{\left(Re_{x}\right)}^{-0.5}$ diminishes. The increment in the nanoparticle diameter originates a regression in both the skin friction coefficients, while a progression in the Nusselt number is observed. The increased diameter of copper nanoparticles results in the reduction of fluid viscosity, ultimately encouraging the nanoliquid’s velocity. Furthermore, the local Nusselt number $Nu_{x}{\left(Re_{x}\right)}^{-0.5}$ increases for higher values of $d_{n}$. The higher values of $s_{p}$ appreciate the heat transfer coefficient.

## 5. Concluding Remarks

This study analyzes impacts of the partial slip, nanoparticle diameter, and nanoparticle shape on the 3-D rotational Maxwell nanofluid $(Cu-H_{2}O$) flow across a linearly stretched surface. The system of underlying PDEs is transformed into dimensionless ODEs via similarity variables. The dimensionless nonlinear ODEs are numerically solved in MATLAB by employing the bvp4c approach. The numerical outcomes for the primary and secondary velocity curves, temperature curve, skin friction coefficients, and Nusselt number are portrayed via graphs and verified via tables. Some of the most significant findings are listed here:

- The growing values of Deborah number β and magnetic constant *M* have declined the velocity $f^{\prime }\left(\xi \right)$ and the amplitude of velocity curve h(ξ), while the progressing trend for the temperature curve θ(ξ) is achieved.
- The velocity along *x* and y–directions is decremented in the vicinity of the sheet for the exceeding rotational effects. An oscillating fashion is examined for larger rotational impacts. Ultimately, the hydraulic boundary layer depth is reduced. The temperature is incremented due to the enhanced kinetic energy of the nanofluid particles for the higher λ values.
- Due to the slip boundary parameter $K_{1}$, both the principle velocity $f^{\prime }\left(\xi \right)$ and the amplitude of secondary velocity h(ξ) decreases while a growing behavior for the nanofluid temperature is observed.
- The improved velocity is induced by a reduction in the viscosity of water-based nanoliquid due to a larger diameter of copper nanoparticles. It is also feasible to get a significant decline in temperature distribution over the region by raising the diameter of copper nanoparticles.
- The increased retardation of nanofluid depreciates the x–component of velocity $f^{\prime }\left(\xi \right)$ as well as the amplitude of the *y*-component of velocity h(ξ) for the addition of higher concentration $\phi_{s}$ of copper nanoparticles. An appreciating trend for θ(ξ) is analyzed.
- The blade shape of copper nanoparticles is proved to be more effective for higher heat transport rates.
- The skin frictions $\left({\left(Re_{x}\right)}^{0.5}\tilde{C}f_{x},{\left(Re_{x}\right)}^{0.5}\tilde{C}f_{y}\right)$ gain lower values for the advancing values of λ,β, and $d_{n}$. A boosting fashion is obtained for the larger values of $K_{1}$, and $\phi_{s}$.
- The wall heat transport coefficient $Nu_{x}{\left(Re_{x}\right)}^{-0.5}$ shows an ascending trend for higher values of nanoparticle diameter $\left(d_{n}\right)$ and shape $\left(s_{p}\right)$. However, a reversing behavior is examined for the higher parametric values of λ, β, *M*, $K_{1}$, and $\phi_{s}$.

The current study reduces to the conventional viscous Newtonian model for β=0. This analysis may be extended for the hybrid-based nanoliquid, Williamson fluid, Jeffrey fluid, Oldroyd-B fluid and other non-Newtonian fluids. 

## Figures and Tables

**Table 1:** Base liquid and nanoparticles’ thermo-physical properties at $25^{\circ }$ [51].

Characteristics	Copper (Cu)	Water ($H_{2}O$)
σ (Ω.m)	5.96 × $10^{7}$	5.5 × $10^{-6}$
k (W/m·K)	401	0.613
$C_{p}$ (J/kg·K)	385	4179
ρ (kg/m${}^{3}$)	8933	997.1

**Table 2:** Comparative results for $-f^{\prime \prime }\left(0\right)$ and $-h^{\prime }\left(0\right)$ for diverse amounts of λ in case of β=0,$K_{1}=0,M=0$.

λ	Wang et al. [56]		Ali et al. [16]		Hussain et al. [42]		Zaimi et al. [57]		Present Outcomes	
	$-f^{\prime \prime }\left(0\right)$	$-h^{\prime }\left(0\right)$	$-f^{\prime \prime }\left(0\right)$	$-h^{\prime }\left(0\right)$	$-f^{\prime \prime }\left(0\right)$	$-h^{\prime }\left(0\right)$	$-f^{\prime \prime }\left(0\right)$	$-h^{\prime }\left(0\right)$	$-f^{\prime \prime }\left(0\right)$	$-h^{\prime }\left(0\right)$
0.0	1.0000	0.0000	1.0000	0.0000	1.0014	0.00000	1.0000	0.0000	1.000483	0.000000
0.2	–	–	–	–	1.03318	0.23856	1.0331	0.2385	1.032664	0.238519
0.4	–	–	–	–	1.01011	0.43193	1.1009	0.4310	1.101120	0.431088
0.5	1.1384	0.5128	1.13844	0.51283	1.13889	0.51832	1.1384	0.5128	1.138478	0.512684
0.6	–	–	–	–	1.17676	0.58742	1.1764	0.5874	1.176356	0.587333
1.0	1.3250	0.8371	1.32501	0.83715	1.32596	0.83725	1.3250	0.8371	1.325027	0.837108
2.0	1.6523	1.2873	1.65232	1.28732	1.65235	1.28726	1.6523	1.2873	1.652351	1.287258
5.0	–	–	2.39026	2.15024	–	–	2.3901	2.1506	2.390139	2.150526

**Table 3:** Numerical findings of $-\theta^{\prime }\left(0\right)$ for diverse Pr inputs in case of $\beta =0,K_{1}=0,M=0$.

λ	Pr = 0.7			Pr = 2.0			Pr = 7.0		
	Ref. [52]	Ref. [56]	Present Outcomes	Ref. [52]	Ref. [56]	Present Outcomes	Ref. [52]	Ref. [56]	Present Outcomes
0.0	0.454	0.455	0.4625	0.911	0.911	0.9111	1.895	1.894	1.8952
0.5	0.389	0.390	0.4129	0.852	0.853	0.8526	1.851	1.850	1.8511
1.0	0.321	0.321	0.3640	0.770	0.770	0.7720	1.788	1.788	1.7876
2.0	0.242	0.242	0.2420	0.638	0.638	0.6461	1.664	1.664	1.6643

**Table 4:** Fluctuation in Nusselt number $\left(Nu_{x}\right)$, skin friction coefficients $\left(\tilde{C}f_{x},\tilde{C}f_{y}\right)$ values for various values of parameters.

λ	β	*M*	$K_{1}$	$\phi_{s}$	$d_{n}$	$s_{p}$	C˜fxRex1/2	C˜fyRex1/2	NuxRex−1/2
0.1	0.2	1.0	0.5	0.05	0.2	3.0	−0.65067	−0.05407	1.30214
0.3							−0.65914	−0.15648	1.27998
0.5							−0.67319	−0.24585	1.24195
1.0							−0.71575	−0.41292	1.11751
1.0	0.0	1.0	0.5	0.05	0.2	3.0	−0.70802	−0.37761	1.14342
	0.4						−0.72304	−0.44709	1.09144
	0.8						−0.73658	−0.51275	1.03800
	1.2						−0.74907	−0.57582	0.98133
1.0	0.2	1.0	0.5	0.05	0.2	3.0	−0.71575	−0.41292	1.11751
		2.0					−0.76868	−0.33217	1.04024
		3.0					−0.81508	−0.27519	0.96197
		4.0					−0.85483	−0.23418	0.88949
1.0	0.2	1.0	0.1	0.05	0.2	3.0	−1.18117	−0.63742	1.47403
			0.3				−0.88707	−0.49844	1.26405
			0.5				−0.71575	−0.41292	1.11751
			0.7				−0.60204	−0.35395	1.00642
1.0	0.2	1.0	0.5	0.05	0.2	3.0	−0.71575	−0.41292	1.11751
				0.10			−0.63744	−0.38135	1.09187
				0.15			−0.58681	−0.35826	1.06654
				0.20			−0.55147	−0.34034	1.04249
1.0	0.2	1.0	0.5	0.05	0.2	3.0	−0.71575	−0.41292	1.11750
					2.2		−1.44491	−0.81254	1.46309
					4.2		−2.43049	−1.34328	1.65391
					6.2		−3.28989	−1.80224	1.74346
1.0	0.2	1.0	0.5	0.05	0.2	3.0	−0.71575	−0.41292	1.11751
						3.7	−0.71575	−0.41292	1.12687
						4.9	−0.71575	−0.41292	1.14205
						5.7	−0.71575	−0.41292	1.15161

## Data Availability

Not applicable.

## Nomenclature

x,y,z	cartesian coordinates (m)	(u, v, w)	constituents of velocity (m/s)
$B_{o}$	applied magnetic field (kg/s${}^{2}$·A)	*T*	fluid’s temperature (K)
$U_{w}$	stretching velocity (m/s)	*k*	heat conductivity (W/m·K)
*a*	stretching rate constant (1/s)	*h*	inter-particle spacing (m)
$d_{n}$	diameter of nanoparticle (m)	$s_{p}$	nanoparticle shape factor
$f^{\prime }\left(\xi \right)$	dimensionless velocity in *x*-direction	θ(ξ)	dimensionless temperature
h(ξ)	dimensionless velocity in *y*-direction	Pr	Prandtl number
*M*	magnetic parameter	$K_{1}$	slip parameter
$C_{p}$	specific heat capacity (J/kg·K)	$\tilde{C}f_{x}$	x–direction skin friction
$\tilde{C}f_{y}$	*y*-direction skin friction	$Nu_{x}$	the local Nusselt number
$\tilde{q_{w}}$	heat flux through sheet (W/m${}^{2}$)	$T_{w}$	temperature at wall (K)
$T_{\infty }$	ambient temperature (K)	$Re_{x}$	Reynolds number
**Greek symbols**			
ρ	density (kg/m${}^{3}$)	μ	viscosity (kg/m·s)
σ	electrical conductance (A${}^{2}$·s${}^{3}$/kg·m${}^{3}$)	$\tilde{\Omega }$	angular velocity (1/s)
$\lambda_{1}$	fluid’s relaxation time (s)	$\phi_{s}$	solid nanoparticle concentration
ξ	dimensionless variable	λ	rotational parameter
β	Deborah number	ν	kinematic viscosity (m${}^{2}$/s)
**Subscripts**			
*f*	base fluid	nf	nanofluid
s	solid nanoparticle		
